# Core and Accessory Genome Comparison of Australian and International Strains of O157 Shiga Toxin-Producing *Escherichia coli*

**DOI:** 10.3389/fmicb.2020.566415

**Published:** 2020-09-04

**Authors:** Alexander Pintara, Amy Jennison, Irani U. Rathnayake, Glen Mellor, Flavia Huygens

**Affiliations:** ^1^Centre for Immunology and Infection Control, Queensland University of Technology, Herston, QLD, Australia; ^2^School of Biomedical Sciences, Faculty of Health, Queensland University of Technology, Brisbane, QLD, Australia; ^3^Public Health Microbiology, Forensic and Scientific Services, Queensland Health, Brisbane, QLD, Australia; ^4^CSIRO Animal, Food and Health Sciences, Archerfield, QLD, Australia

**Keywords:** STEC, genomics, O157, Australia, epidemiology

## Abstract

Shiga toxin-producing *Escherichia coli* (STEC) is a foodborne pathogen, and serotype O157:H7 is typically associated with severe disease. Australian STEC epidemiology differs from many other countries, as severe outbreaks and HUS cases appear to be more often associated with non-O157 serogroups. It is not known why Australian strains of O157 STEC might differ in virulence to international strains. Here we investigate the reduced virulence of Australian strains. Multiple genetic analyses were performed, including SNP-typing, to compare the core genomes of the Australian to the international isolates, and accessory genome analysis to determine any significant differences in gene presence/absence that could be associated with their phenotypic differences in virulence. The most distinct difference between the isolates was the absence of the *stx2a* gene in all Australian isolates, with few other notable differences observed in the core and accessory genomes of the O157 STEC isolates analyzed in this study. The presence of *stx1a* in most Australian isolates was another notable observation. Acquisition of *stx2a* seems to coincide with the emergence of highly pathogenic STEC. Due to the lack of other notable genotypic differences observed between Australian and international isolates characterized as highly pathogenic, this may be further evidence that the absence of *stx2a* in Australian O157 STEC could be a significant characteristic defining its mild virulence. Further work investigating the driving force(s) behind Stx prophage loss and acquisition is needed to determine if this potential exists in Australian O157 isolates.

## Introduction

Shiga toxin-producing *Escherichia coli* (STEC) is a foodborne human pathogen that causes a wide spectrum of disease varying in severity from asymptomatic carriage, to haemorrhagic colitis, through to life threatening disease such as haemolytic uremic syndrome (HUS) (Paton and Paton, 1998; Castro et al., 2017; Kim et al., 2020). This wide spectrum of disease caused by STEC is due, in part to the immune competence of the individual affected (Karmali et al., 2003), and to the genetic heterogeneity found within STEC strains which involves several virulence factors where their presence/absence or variation within a strain can affect the severity of disease caused by the strain (Bugarel et al., 2011; Haugum et al., 2014). The pathogenesis of STEC is therefore complex and not well understood, where exploration of strains differing in virulence may elucidate key virulence factors responsible for severe disease.

Shiga toxin-producing *Escherichia coli* was first recognized as an emerging pathogen following the investigation of two haemorrhagic colitis outbreaks in 1982, leading to the identification of *E. coli* serotype O157:H7 as the cause (Riley et al., 1983). This led to the association of the O157:H7 serotype of *E. coli* with STEC. Since 1982 many other serotypes of *E. coli* have been associated with STEC (Hughes et al., 2006), yet O157:H7 remains the most common outbreak- and severe disease-associated serotype in many developed nations (Rangel et al., 2005; Werber et al., 2008; Terajima et al., 2014). Australian STEC epidemiology differs from many other countries, as severe outbreaks and HUS cases appear to be more often associated with non-O157 serogroups (Paton et al., 1996, 1999; McPherson et al., 2009; Vally et al., 2012). Australian O157 STEC strains are also predominantly non-motile, therefore having an O157:H- serotype, and this has been associated with a single insertion in the *flgF* gene (Pintara et al., 2018). This is exemplified by two of Australia’s largest outbreaks; an outbreak that occurred in 1995 in South Australia, and a 2013 outbreak that was associated with an annual agricultural show in Queensland (Centers for Disease Control and Prevention, 1995; Vasant et al., 2017). The cause of the 1995 outbreak was found to be mostly due to O111:H- STEC, where 23 of the 51 cases developed HUS. Conversely, for the 2013 outbreak, which was due to O157:H- STEC, none of the 57 cases developed HUS and was associated with a lower hospitalization rate than what is typically associated with STEC outbreaks. It is not known why there are differences in virulence between the Australian O157 STEC and international strains but studies have demonstrated some genotypic differences, notably the absence of *stx2a* (Mellor et al., 2012; Ingle et al., 2019).

An interesting genomic comparison to make would be to compare Australian strains with a United Kingdom clone that was responsible for an outbreak in 2015. Interestingly, this clone had emerged from an O157 strain that was previously characterized as being mild and not commonly associated with outbreaks (Byrne et al., 2018). It was reported as having acquired a different Shiga toxin profile through the loss of one subtype of the Shiga toxin genes, and the later acquisition of another. Shiga toxins are the major virulence factor associated with STEC, where different subtypes of Shiga toxin genes (*stx*) have been associated with different levels of virulence (Fuller et al., 2011). As this United Kingdom clone became highly pathogenic by acquiring a different Shiga toxin profile to the mild progenitor strain, the underlying genetic background necessary for high pathogenicity may be present within this strain.

We have shown that an O157 clone prevalent in Australia clinical cases contains a unique single base insertion potentially responsible for its non-motile phenotype (Pintara et al., 2018), and in this study, we seek to improve our understanding of genotypes associated with different incidences and severity of disease by comparing this Australian O157 clone with strains related to the United Kingdom 2015 outbreak and additional international O157 STEC strains. Multiple genetic analyses were performed to determine differences between the core and accessory genomes of Australian and international STEC strains. The aim of the analysis was to identify molecular determinants for virulence in STEC and their association with Australian O157 strains.

## Materials and Methods

### Isolate Selection

A total of 164 isolates were included in the analysis, including 76 Australian isolates from between 1986 to 2018 obtained from various sources to be representative of the Australian geographic region (Supplementary Table 1), and 68 isolates from the United Kingdom 2015 outbreak (Byrne et al., 2018) obtained from the National Center for Biotechnology Information (NCBI) Short Read Archive. An additional 20 international STEC genomes were also obtained from the NCBI GenBank^1^ and other sources (Table 1).

**TABLE 1:** International O157 STEC strains used in the analysis.

Isolate	Isolation year	Location	Source	Obtained from	Accession number
155	2012	United Kingdom	Human	NCBI GenBank	CP018237.1
272	2013	United Kingdom	Human	NCBI GenBank	CP018239.1
319	2012	United Kingdom	Human	NCBI GenBank	CP018241.1
350	2011	United Kingdom	Human	NCBI GenBank	CP018243.1
EC4115	2006	United States	Human	NCBI GenBank	CP001164.1
EDL933	1982	United States	Ground beef	NCBI GenBank	CP008957.1
FRIK2069	2011	United States	Cattle	NCBI GenBank	CP015846.1
FRIK944	2014	United States	Cattle	NCBI GenBank	CP016625.1
JEONG-1266	2013	United States	Cattle	NCBI GenBank	CP014314.1
M73607	2009	Japan origin	Human	This study	SRR12398594
M74957	2014	New Zealand origin	Human	(Pintara et al., 2018)	SAMEA104371134
M76457	2012	Denmark origin	Human	This study	SRR12398593
M76948	2013	Turkey origin	Human	(Pintara et al., 2018)	SAMEA104371139
M77548	2015	Unknown travel history outside of Australia	Human	(Pintara et al., 2018)	SAMEA104371140
M78364	2015	Travelers diarrhea outside of Australia	Human	This study	SRR12398592
Sakai	1996	Japan	Human	NCBI GenBank	BA000007.3
SS17	2014?	United States	Cattle	NCBI GenBank	CP008805.1
SS52	2014?	United States	Cattle	NCBI GenBank	CP010304.1
TW14359	2006	United States	Human	NCBI GenBank	CP001368.1
Xuzhou21	1999	China	Human	NCBI GenBank	CP001925.1

### Whole Genome Sequencing

Whole genome sequencing of all the study isolates was done using previously described methods (Pintara et al., 2018).

QIAamp DSP DNA mini kit (Qiagen, Germany) was used to extract DNA from the isolates using the QIAsymphony SP, following the Tissue HC 200 V DSP protocol. Quantitation of extracted DNA was done on a plate reader using the Quant-IT kit (Thermo Fisher Scientific, United States).

The Nextera XT DNA library preparation kit (Illumina, United States) was used for library preparation of the DNA samples. The DNA samples were then sequenced on the NextSeq500 (Illumina, United States) using the NextSeq 500 Mid Output V2 kit (Illumina, United States).

Trimmomatic v0.36 was used to trim sequence reads from the sequenced isolates and quality checked by FastQC v0.11.5 (Andrews, 2014; Bolger et al., 2014). Trimmed reads were then *de novo* assembled using the SPAdes assembler version 3.9.1 (Bankevich et al., 2012) and assemblies annotated using Prokka (Overbeek et al., 2014).

### Core Genome Analysis

Single nucleotide polymorphisms (SNP) in the core genome (homologous sequences conserve in all aligned genomes included in this study) were identified using Snippy (v3.0)^2^, using the AUSMDU00002545 genome as a reference. SNPs from Snippy results of all genomes were aligned as a core SNP alignment and used to generate a hierarchal cluster tree. The tree was constructed using average clustering on the Jaccard distance of SNP differences between the 164 STEC O157 strains obtained from NCBI GenBank and Short Read Archive, and the sequenced genomes. Bootstrapping at 10,000 iterations was used to assess the certainty of clusters using the R package, pvclust (Suzuki and Shimodaira, 2006).

### Accessory Genome Analysis

All assembled genomes, including annotated genomes obtained from NCBI GenBank, were annotated using Prokka version 1.13 to avoid artifacts potentially produced due to genes being annotated differently by different annotation programs. The general feature format (gff) files produced by Prokka were used to make comparisons between all isolates to identify the accessory genome using Roary with the option to not spilt clusters containing paralogs (Page et al., 2015). The pan genome reference FastA file produced by Roary, which lists all gene sequences present in all genomes analyzed, was then used to validate the Roary results by mapping sequence reads to the pan genome reference FastA file using CLC genomics version 11.0.1. Using a gene coverage cut-off of 99% and identifying genes with frameshifting indel mutations as not present, the accessory genome of all selected isolates was identified as genes not present in all genomes analyzed.

### Virulome Analysis

Gene selection for the STEC virulome analysis was done after reviewing the literature of virulence factors associated with virulence in STEC (Supplementary Table 2, including references). As with the accessory genome analysis, reference genes for the virulence factors were obtained from NCBI GenBank and used to map sequence reads from all selected isolates. Using a gene coverage cut-off of 99% and identifying genes with frameshifting indel mutations as not present, the virulome of all selected isolates was determined.

### Statistics

Pairwise comparisons between Australian clinical and United Kingdom 2015 outbreak isolates were done using Chi-square tests to investigate significant genetic differences in core genome SNPs and accessory genes between the two groups. Microsoft Excel was used to calculate *P*-values which were then adjusted with a Bonferroni correction. Any genetic differences with an adjusted *P*-value < 0.05 were considered as significant.

## Results and Discussion

### Core Genome Analysis

**FIGURE 1:**
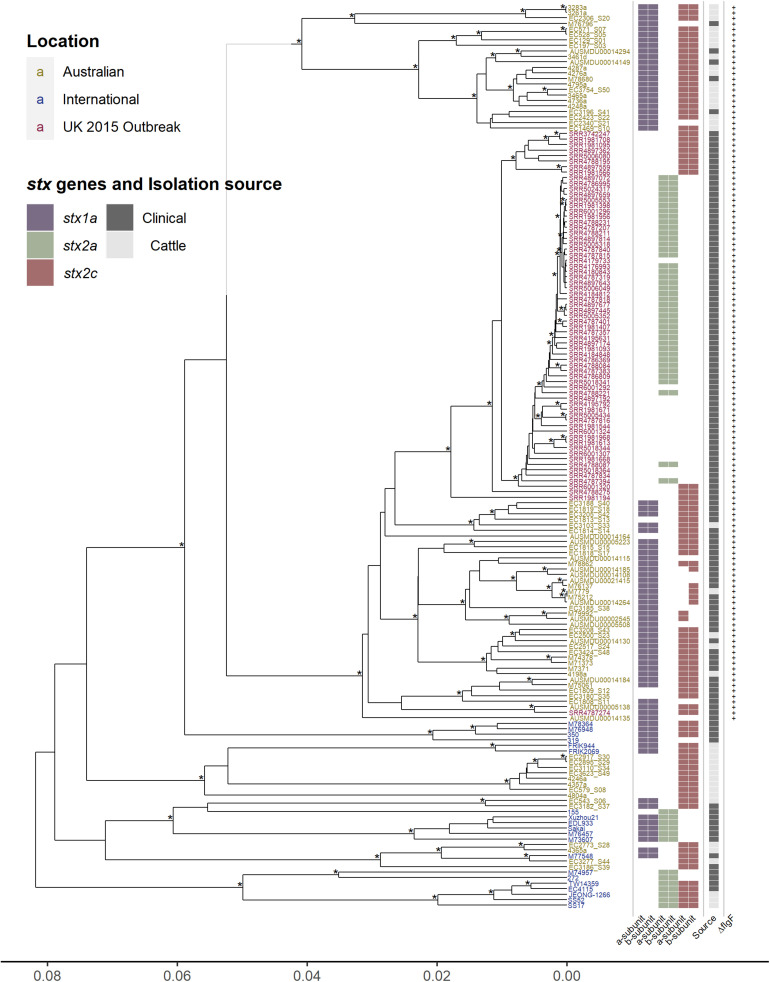
Hierarchal cluster tree of serogroup O157 STEC genomes based on the Jaccard distance of SNP differences identified by mapping all isolates to the AUSMDU00002545 genome as a reference. Asterisk indicates bootstrap support of >95%. Isolates labeled in yellow are Australian, isolates in blue are international, and isolates in red are associated with the United Kingdom 2015 outbreak. Indicated to the right of the tree are; the subtype of both the A and B subunits of the Shiga toxin genes, purple for *stx1a*, green for *stx2a*, and red for *stx2c*; whether the isolates were clinically isolated (dark gray), or from cattle (light gray); and whether the isolates carry the single cytosine base inserted at 125 nt of the *flgF* gene, indicated by the presence of a plus sign.

The unique epidemiology of Australian O157 STEC has been associated with mild disease and is not typically associated with outbreaks (Paton et al., 1996, 1999; McPherson et al., 2009; Vally et al., 2012), contrasting with the epidemiology more typically observed internationally (Rangel et al., 2005; Werber et al., 2008; Terajima et al., 2014). To determine the genetic characteristics associated with this contrasting epidemiology, core genome comparisons were made between 164 O157 isolates (76 Australian, 20 international, and 68 United Kingdom 2015 outbreak isolates), analyzing regions of the genome that are shared amongst all 164 O157 isolates. The genetic relationship between the isolates were visualized by constructing a hierarchal cluster tree using the pairwise differences in core genome SNPs (Figure 1). The Australian isolates, which carry the single base insertion in the *flgF* gene, cluster separately from the international isolates, where, within this cluster, most Australian cattle isolates form a separate sub-cluster from the Australian clinical isolates. Surprisingly, the United Kingdom 2015 isolates appear to be most closely related to the Australian clinical isolates, rather than to any of the international isolates. All United Kingdom isolates also carry the single cytosine base insertion in *flgF* at 125nt, a feature described previously for Australian O157 STEC (Pintara et al., 2018; Ingle et al., 2019). The apparent phylogenetic similarity between the Australian clinical and United Kingdom isolates as shown in Figure 1, despite their geographical difference, may be likely due to the lineage shared by the strains. Phylogenetic analysis has shown that Australian isolates characterized with the insertion in *flgF* are typically of lineage II (Ingle et al., 2019), likewise with the United Kingdom isolates selected to be analyzed in this study (Byrne et al., 2018).

Given the core genetic similarity, yet differences in reported virulence between the Australian and United Kingdom outbreak clone, further accessory genome analysis was performed to identify any genetic determinants possibly responsible for the differences in the pathogenic phenotype. Any genetic differences between these strains would more likely explain the differences in pathogenic phenotype rather than comparing Australian clinical with any other more distantly related sets of isolates as there would be fewer differences that are inconsequential to virulence.

Significant SNP differences between the clinical Australian isolates and the United Kingdom isolates were determined by doing a pairwise comparison of the SNP differences found in the core genome (Supplementary Table 3). The analysis found 141 significant SNP differences (*P* adjusted value < 0.05), from the 2,794 core genome SNPs found between the Australian clinical and United Kingdom isolates. Out of these 141 significant SNPs, 27 were intergenic, 41 were synonymous, 69 were non-synonymous, and there were 4 stop gained/lost SNPs. Of interest was a non-synonymous mutation in the hemolysin B gene, *hlyB*/*ehxB*, found significantly present in the United Kingdom isolates (*P* adjusted value < 0.0001). There were two stop gained mutations found significantly present in the United Kingdom isolates (*P* adjusted value < 0.0001): one in the putrescine-ornithine antiporter gene, *potE*, which has been potentially linked as a regulatory factor for ornithine-dependent acid resistance (Aquino et al., 2017); and the other in a hypothetical gene. There were also two stop gained mutations found significantly present in the Australian clinical isolates (*P* adjusted value < 0.001). This included the urease accessory protein gene, *ureD*, and in a hemagglutinin/hemolysin-related gene. It is difficult to speculate how these genetic differences may be responsible for virulence differences in these isolates, however, one distinct difference with obvious relevance to virulence is seen across all isolates in Figure 1, the Shiga toxin profile.

Distinct differences in Shiga toxin profiles were observed, with most of the international and United Kingdom isolates carrying the *stx2a* subtype of the Shiga toxin gene, whereas none of the Australian isolates had this subtype. The *stx* subtypes found amongst Australian isolates was *stx1a* in combination with *stx2c* (45/76), *stx1a* on its own (17/76), and *stx2c* on its own (14/76) (Figure 1). Investigations of the insertion sites of Stx-phages in Australian clinical and cattle O157 isolates typically show a AS12c SBI genotype, with *argW* occupied by *stx1a*-phage and *sbcB* by an *stx2c*- (Mellor et al., 2012; Jaros et al., 2014). The *stx2a* subtype is typically absent in Australian O157 strains (Mellor et al., 2012; Ingle et al., 2019). It has previously been reported that the United Kingdom 2015 outbreak was associated with high pathogenicity, and proposed that this was caused by the emergence of a clone that evolved from a STEC O157:H7 *stx-*negative ancestor after it acquired a bacteriophage encoding Shiga *stx2a*, which in turn had evolved from a *stx2c* progenitor (Byrne et al., 2018). The Stx2a subtype has been reported to be most commonly associated with severe disease in humans, while Stx2c and Stx1a much less (Ostroff et al., 1989; Boerlin et al., 1999; Orth et al., 2007; Kawano et al., 2012; Marejková et al., 2013; Brandal et al., 2015; Legros et al., 2018).

Another distinct feature is the presence of *stx1a* in most of the Australian isolates. Stx1a has been shown to have antagonistic effects against Stx2a toxicity, shown in both *in vitro* and *in vivo* models, where Stx2a alone appears to be more potent (Petro et al., 2019). This reduction in Stx2a toxicity is potentially due to the stronger receptor binding affinity to the globotriaosylceramide (Gb3) receptor of the B subunit of Stx1a, blocking out the binding of Stx2a to the Gb3 receptor (Head et al., 1991; Tesh et al., 1993; Zumbrun et al., 2010; Karve and Weiss, 2014; Russo et al., 2014; Cherubin et al., 2019). The reduced toxicity of Stx2a in the presence of Stx1a has also been linked epidemiologically, reporting a reduced risk of HUS from STEC strains that possess an *stx1a* and *stx2a* genotype (Ostroff et al., 1989; Brandal et al., 2015; Tarr et al., 2019). The presence of two or more *stx*-encoding prophage regions expressing different subtypes of Shiga toxins may not necessarily cause an additive potential in virulence, the effects can be synergistic or antagonistic. Therefore, the presence of *stx1a* may be an important distinction to consider in relation to Australian O157 STEC. The acquisition of *stx2a* in Australian isolates may not result in a similar pathogenic phenotype as described with the United Kingdom isolates. It may be somewhat attenuated in comparison as epidemiological observations have reported that *stx2a*-only strains are more strongly associated with HUS than strains that possess both *stx1a* and *stx2a*. The possible competitive inhibitory effect of Stx1a against Stx2a is a potential explanation of this epidemiological observation.

### Virulome Analysis

**FIGURE 2:**
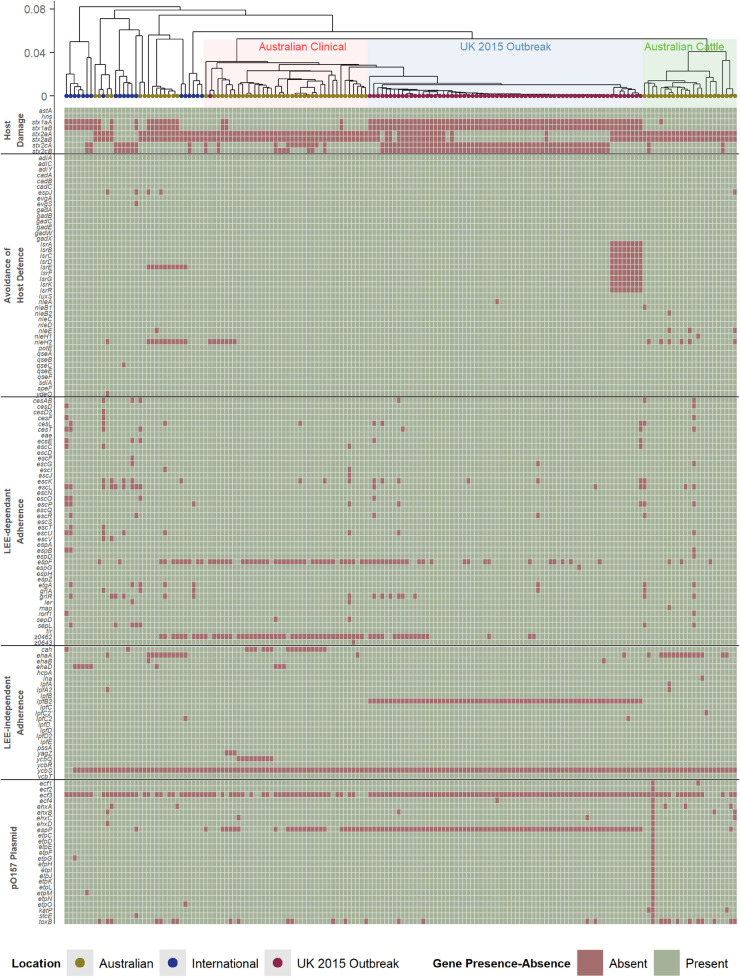
Hierarchal cluster tree as shown in Figure 1, highlighting the Australian clinical isolates in red and United Kingdom outbreak isolates in blue. Below the tree is a heatmap indicating the presence (green) and absence (red) of genes reported to be associated with virulence in STEC. The virulence genes have been categorized into difference pathogenic processes involved in STEC pathogenesis.

While the presence of Shiga toxin genes is a key and essential component of STEC, which are typically integrated in the *E. coli* genome as part of a prophage region, other genetic factors are important in considering the pathogenic potential of STEC strains. The broad clinical spectrum seen in STEC disease in humans is partially due to variation in the presence, absence, and variation in virulence factors which contributes to complex pathogenesis. The pathogenesis of STEC is not comprehensively understood but typically involves the colonization of mucosal sites, avoidance of host defense mechanisms, and host damage (Nataro and Kaper, 1998; Farfan and Torres, 2012; Ho et al., 2013; Franzin and Sircili, 2015; Kendall and Sperandio, 2016; Kim et al., 2016). These genetic factors, which can contribute to the pathogenicity of a strain, have been well-reported for the O157 serogroup of STEC (Supplementary Table 2). In this study, these factors (Figure 2) were used to analyze the O157 STEC virulome to determine if there are any other potential underlying differences that could explain the pathogenic differences amongst the Australian clinical, with the international and United Kingdom isolates.

Australian O157 STEC appear to have a typical O157 virulome also found in the United Kingdom and international isolates analyzed in this study. This includes the presence of most Locus of Enterocyte Effacement (LEE)-dependant and -independent factors. STEC associated with severe human disease are capable of colonizing intestinal mucosal sites, and the main mechanism reported to facilitate this in O157 STEC is the LEE Pathogenicity Island (LEE PAI), a common genetic background trait in O157 STEC (Perna et al., 1998). Many LEE-independent factors are also able to facilitate intestinal colonization (Farfan and Torres, 2012), and many of these factors reported to be found in O157 STEC (Coombes et al., 2008), are also present in the Australian clinical isolates. Another common trait amongst pathogenic O157 STEC is the presence of the putative virulence plasmid, pO157 (Lim et al., 2010). The plasmid appears to be present in Australian clinical isolates, with no distinct differences found amongst the genes that are carried by the plasmid. Avoidance of host defense mechanisms is another important factor in helping STEC to establish an infection, where this includes acid resistance mechanisms, methods to regulate gene expression in response to stressful environmental host conditions, and modulation of host immune cell response (Large et al., 2005; Yen et al., 2016; Park et al., 2017). Likewise, no discernible differences were found. The only distinct difference found was the absence of both subunits of *stx1a* in most international and United Kingdom isolates, while its presence is noted in almost all Australian isolates (Figure 1). There does not appear to be any additional differences between typical O157 virulence factors that associated with the difference in Australian STEC pathogenicity compared to international O157 STEC isolates, except for the *stx* profile.

### Accessory Genome Analysis

**FIGURE 3:**
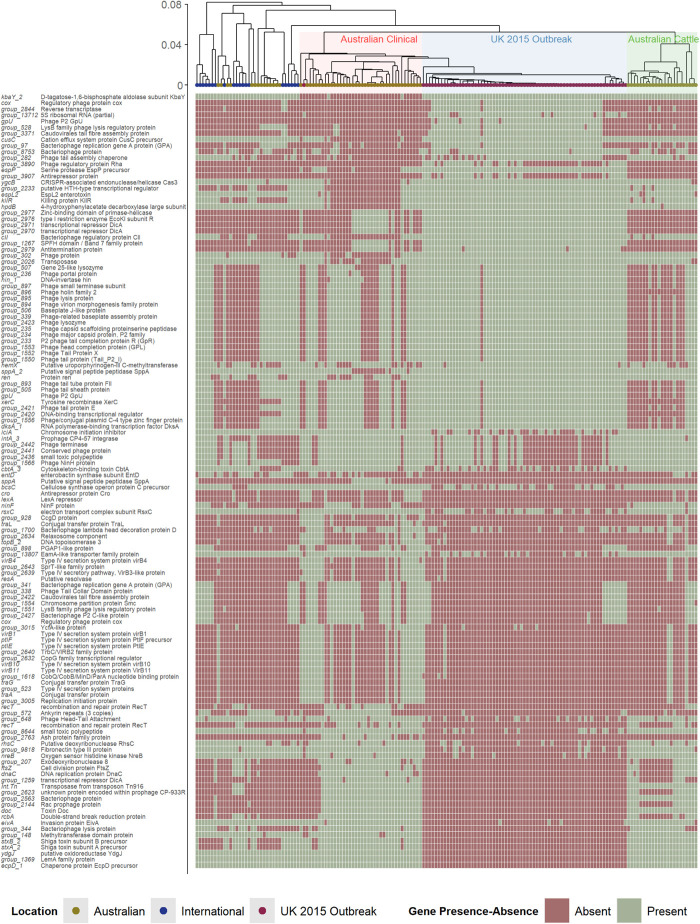
Hierarchal cluster tree as shown in Figure 1, highlighting the Australian clinical isolates in red and United Kingdom outbreak isolates in blue. Below the tree is a heatmap indicating the presence (green) and absence (red) of accessory genes found to be significantly different in their presence and absence between Australian clinical and United Kingdom outbreak isolates.

To consider the role of genes beyond those reported to be involved directly in virulence, the entire accessory genomes (<100% of genes shared amongst all strains) of the Australian clinical and United Kingdom isolates was analyzed by pairwise comparison (Figure 3). The heatmap in Figure 3 shows the presence/absence of accessory genes that were found to be significantly different (*P* value < 0.05) between Australian clinical and United Kingdom isolates (Supplementary Table 4). In total, 127/5,427 significant differences (excluding hypothetical genes) were found between the Australian clinical and United Kingdom isolates when comparing the presence/absence of accessory genes. Many of the genes were bacteriophage derived, however, there were a few genes with obvious relevance to virulence. This included many type IV secretion system genes found to be significantly present in the clinical Australian isolates, and a type III secretion system gene, *eivA*, reported to be involved in invasion (Yao et al., 2009), significantly present in the Australian clinical isolates as well. It is difficult to speculate how the presence of these genes in Australian clinical isolates would attenuate the pathogenicity of these strains, perhaps these genes benefit the overall fitness of these isolates within the Australian niche. The differences found in bacteriophage derived genes, however, requires further investigation.

The pathogenic potential of *stx2a*-producing strains can differ due to differences in Stx-production levels, and this difference can be due to differences in bacteriophage genes that make up the prophage structure (Ogura et al., 2015). An interesting feature to note about the United Kingdom 2015 outbreak isolates is that the prophage region containing the *stx2a* gene has a very similar structure to the *stx2c* prophage and is inserted in the *sbcB* SBI site, the typical *stx2c*-phage integration site (Yara et al., 2019). The *stx2c*-prophage structure tends to be homologous and associated with low expression of *stx*, whereas *stx2a*-prophage structure is found to be highly heterogeneous and associated with high variability in *stx* expression. It was reported that, while the *stx2a*-carrying United Kingdom strains were more pathogenic than isolates that possessed *stx2c* alone or that were negative for *stx*, there were no reported cases of HUS. The fact that the *stx2c*-prophage is associated with low expression of *stx* may have potentiated the lack of reported HUS cases with the *stx2a*-carrying strains.

It is also important to consider what effect other prophage regions in STEC isolates may cause in relation to pathogenicity. The study of bacteriophage derived genes and the prophage regions they form within the host was beyond the scope of this study as a major limitation was that most of the isolates included in the analysis have been short-read sequenced. Assemblies of short-read sequenced isolates are known to produce highly fragmented assembled genomes, where many genes can fail to be assembled properly, being split across multiple contigs (Schmid et al., 2018), particularly where many of these genes are typically bacteriophage-related (Klumpp et al., 2012). Lambdoid bacteriophages, temperate bacteriophages that lysogenize by site-specific recombination with the bacterial chromosome, make up a large part of STEC genomes (Canchaya et al., 2003), expressing many similar regulatory factors. This may facilitate crosstalk amongst prophage regions potentially affecting the expression of phage-related genes, such as *stx* (Serra-Moreno et al., 2008). The phage-host relationship is something else to consider, which represents a complex interaction between phage regulatory genes and host factors; where this interaction may affect the expression of host derived factors (Berger et al., 2019).

Another further limitation of this study is that gene regulatory factors such as promoter regions and transcription factor binding sites were not analyzed. Disruption to regulatory regions can affect the expression of associated genes, ultimately affecting the phenotype of the organism. Regulatory regions associated with virulence genes are therefore important factors to analyze as disruptions to these regions can affect the virulence of a strain and therefore, should not be overlooked. RNAseq of O157 STEC may be utilized to determine if differences in the expression of virulence genes between isolates exist, which then can help to provide clues of any potential disruption to associated regulatory regions.

## Conclusion

The most distinct difference in relation to virulence between the Australian STEC isolates was the absence of *stx2a*, with other notable differences including the presence of many type IV secretion system genes and differences in bacteriophage-derived genes observed in the core and accessory genomes of the O157 STEC isolates analyzed in this study. The absence of *stx2a* has been reported in other studies describing the genotype of Australian O157 STEC isolates (Mellor et al., 2012; Ingle et al., 2019), and the lack of other virulence-related genotypic differences found between the Australian with the international and United Kingdom O157 isolates in the present study, suggests that the absence of *stx2a* is associated with a mild virulence phenotype. As has been described with the emergence of other highly pathogenic STEC (Xiong et al., 2012; Bielaszewska et al., 2013; Byrne et al., 2018), the acquisition of *stx2a* in a previously characterized mild strain seems to coincide with its emergence as a highly pathogenic pathogen. With the lack of notable genotypic differences described here, there may exist a similar potential for Australian O157 STEC if a clone were to acquire *stx2a*. Understanding how the loss and acquisition of Stx prophage occurs and the driving force(s) behind this loss and acquisition, could be used to prevent a similar occurrence in Australia through improved surveillance, resulting in big industry and public health impacts in terms of reducing economic impact and improving disease prevention. In this study we have shown that the isolates related to a highly pathogenic outbreak in the United Kingdom were most closely related to isolates from Australian cases, rather than to any of the other international isolates included in the analysis. Further exploration of the phylogenetics between Australian and these United Kingdom isolates could be important to see if the loss and acquisition of Stx prophage in United Kingdom isolates could be replicated in Australian isolates, determining if this potential to change Shiga toxin profiles exist for Australian O157 STEC.

## Data Availability Statement

The datasets presented in this study can be found in online repositories. The names of the repository/repositories and accession number(s) can be found in the article/Supplementary Material.

## Author Contributions

All authors contributed to the conceptualization of the study, and the review and editing of the manuscript. AP performed the data curation, analysis, and writing of initial draft of the article. AP, AJ, and IR were involved in the investigation and methodology. AJ, IR, GM, and FH helped to provide the resources and supervision for the study. AJ and FH acquired funding. FH was the project administer.

## Conflict of Interest

The authors declare that the research was conducted in the absence of any commercial or financial relationships that could be construed as a potential conflict of interest.

## Footnotes

1 https://www.ncbi.nlm.nih.gov/genbank/

2 https://github.com/tseemann/snippy
