# Supplementary material for: Core and Accessory Genome Comparison of Australian and International Strains of O157 Shiga Toxin-Producing Escherichia coli
Source: Front Microbiol. 2020 Sep 4;11:566415. doi: 10.3389/fmicb.2020.566415 (PMC7498637; doi:10.3389/fmicb.2020.566415)
Supplement: Supplementary file 1 [file Table_1.DOCX]

**Supplementary Table 1.** Australian O157 STEC Isolate selection

| Isolate | Isolation Year | Source | Obtained from | Database acquisition number |
| --- | --- | --- | --- | --- |
| 3261a | 2009 | Cattle | This study | SRR12398601 |
| 3283a | 2009 | Cattle | This study | SRR12398600 |
| 3461d | 2012 | Cattle | This study | SRR12398591 |
| 3465a | 2012 | Cattle | This study | SRR12398590 |
| 4198a | 2014 | Cattle | This study | SRR12398589 |
| 4246a | 2014 | Cattle | This study | SRR12398588 |
| 4248a | 2014 | Cattle | This study | SRR12398587 |
| 4276a | 2014 | Cattle | This study | SRR12398586 |
| 4287a | 2015 | Cattle | This study | SRR12398585 |
| 4357a | 2015 | Cattle | This study | SRR12398584 |
| 4365a | 2015 | Cattle | This study | SRR12398599 |
| 4736a | 2017 | Cattle | This study | SRR12398598 |
| 4795a | 2017 | Cattle | This study | SRR12398597 |
| 4804a | 2018 | Cattle | This study | SRR12398596 |
| AUSMDU00002545 | 2013 | Clinical | (1) | CP045975.1 |
| EC129_S01 | 1996 | Cattle | (2) | SAMN09981727 |
| EC1469_S10 | 1998 | Cattle | (2) | SAMN09981728 |
| EC1808_S11 | 1991 | Clinical | (2) | SAMN09981729 |
| EC1809_S12 | 1991 | Clinical | (2) | SAMN09981730 |
| EC1813_S13 | 1987 | Clinical | (2) | SAMN09981731 |
| EC1814_S14 | 1988 | Clinical | (2) | SAMN09981732 |
| EC1815_S15 | 1997 | Clinical | (2) | SAMN09981733 |
| EC1818_S17 | 1986 | Clinical | (2) | SAMN09981735 |
| EC1819_S18 | 1999 | Clinical | (2) | SAMN09981736 |
| EC197_S03 | 1996 | Cattle | (2) | SAMN09981738 |
| EC2306_S20 | 2002 | Cattle | (2) | SAMN09981739 |
| EC2340_S21 | 2002 | Cattle | (2) | SAMN09981740 |
| EC2423_S22 | 2002 | Cattle | (2) | SAMN09981741 |
| EC2500_S23 | 2002 | Cattle | (2) | SAMN09981742 |
| EC2517_S24 | 2003 | Cattle | (2) | SAMN09981743 |
| EC2773_S28 | 2004 | Cattle | (2) | SAMN09981744 |
| EC2895_S29 | 2005 | Cattle | (2) | SAMN09981745 |
| EC2917_S30 | 2005 | Cattle | (2) | SAMN09981746 |
| EC3103_S33 | 2008 | Cattle | (2) | SAMN09981748 |
| EC3110_S34 | 2009 | Cattle | (2) | SAMN09981749 |
| EC3180_S35 | 1996 | Clinical | (2) | SAMN09981750 |
| EC3182_S37 | 1999 | Clinical | (2) | SAMN09981752 |
| EC3185_S38 | 2000 | Clinical | (2) | SAMN09981753 |
| EC3186_S39 | 2000 | Clinical | (2) | SAMN09981754 |
| EC3188_S40 | 2002 | Clinical | (2) | SAMN09981755 |
| EC3196_S41 | 2004 | Clinical | (2) | SAMN09981756 |
| EC3205_S42 | 2008 | Clinical | (2) | SAMN09981757 |
| EC3208_S43 | 2009 | Clinical | (2) | SAMN09981758 |
| EC3277_S44 | 2009 | Cattle | (2) | SAMN09981759 |
| EC3424_S48 | Unknown | Clinical | (2) | SAMN09981762 |
| EC3623_S49 | 2013 | Cattle | (2) | SAMN09981763 |
| EC3754_S50 | 2013 | Cattle | (2) | SAMN09981764 |
| EC528_S05 | 1993 | Cattle | (2) | SAMN09981765 |
| EC543_S06 | 1997 | Cattle | (2) | SAMN09981766 |
| EC571_S07 | 1993 | Cattle | (2) | SAMN09981767 |
| EC579_S08 | 1994 | Cattle | (2) | SAMN09981768 |
| M71373 | 2016 | Clinical | (3) | SAMEA104371132 |
| M7371 | 2017 | Clinical | (3) | SAMEA104371143 |
| M74378 | 2016 | Clinical | (3) | SAMEA104371133 |
| M75061 | 2015 | Clinical | (3) | SAMEA104371135 |
| M75212 | 2013 | Clinical | (3) | SAMEA104371136 |
| M76137 | 2011 | Clinical | (3) | SAMEA104371137 |
| M76796 | 2015 | Clinical | (3) | SAMEA104371138 |
| M7779 | 2013 | Caprine | This study | SRR12398595 |
| M78680 | 2016 | Clinical | (3) | SAMEA104371141 |
| M78862 | 2015 | Clinical | (3) | SAMEA104371142 |
| M79992 | 2016 | Clinical | (3) | SAMEA104371144 |
| AUSMDU00005223 | 2007-2016 | Clinical | (4) | SRR8592091 |
| AUSMDU00005508 | 2007-2016 | Clinical | (4) | SRR8592113 |
| AUSMDU00005138 | 2007-2016 | Clinical | (4) | SRR8592172 |
| AUSMDU00021415 | 2007-2016 | Clinical | (4) | SRR8592202 |
| AUSMDU00014135 | 2007-2016 | Clinical | (4) | SRR8625648 |
| AUSMDU00014149 | 2007-2016 | Clinical | (4) | SRR8625652 |
| AUSMDU00014130 | 2007-2016 | Clinical | (4) | SRR8625712 |
| AUSMDU00014264 | 2007-2016 | Clinical | (4) | SRR8625718 |
| AUSMDU00014115 | 2007-2016 | Clinical | (4) | SRR8625745 |
| AUSMDU00014108 | 2007-2016 | Clinical | (4) | SRR8625747 |
| AUSMDU00014185 | 2007-2016 | Clinical | (4) | SRR8625830 |
| AUSMDU00014294 | 2007-2016 | Clinical | (4) | SRR8625896 |
| AUSMDU00014184 | 2007-2016 | Clinical | (4) | SRR8625897 |
| AUSMDU00014164 | 2007-2016 | Clinical | (4) | SRR8625904 |
