# Supplementary material for: Core and Accessory Genome Comparison of Australian and International Strains of O157 Shiga Toxin-Producing Escherichia coli
Source: Front Microbiol. 2020 Sep 4;11:566415. doi: 10.3389/fmicb.2020.566415 (PMC7498637; doi:10.3389/fmicb.2020.566415)
Supplement: Supplementary file 2 [file Table_2.DOCX]

**Supplementary Table 2.** Virulence genes used in virulome analysis

| Host Damage | | | | | |
| --- | --- | --- | --- | --- | --- |
| Virulence factor | **Gene** | **Role** | **Genome POS** | **Accession No.** | **Reference** |
| EAST1 toxin | astA | Enterotoxin | 2334518-2335552 | CP001368.1 | (5) |
| DNA-binding protein | hns | Repression of Enterohaemolysin | 1829490-1829903 | AE005174.2 | (6) |
| Shiga toxin | stx1A | Cytotoxin | 2996033-2996980 | AE005174.2 | (7) |
| Shiga toxin | stx1B | Cytotoxin | 2995754-2996023 | AE005174.2 | (7) |
| Shiga toxin | stx2A | Cytotoxin | 1352290-1353249 | AE005174.2 | (7) |
| Shiga toxin | stx2B | Cytotoxin | 1353261-1353530 | AE005174.2 | (7) |
| Avoidance of Host Defence | | | | | |
| Virulence factor | **Gene** | **Role** | **Genome POS** | **Accession No.** | **Reference** |
| Arginine-dependent acid resistance system (AR3) | adiA | Acid resistance | 5223964-5226234 | CP001368.1 | (8) |
| Arginine-dependent acid resistance system (AR3) | adiC | Acid resistance | 5221404-5222741 | CP001368.1 | (8) |
| Arginine-dependent acid resistance system (AR3) | adiY | Acid resistance | 5222878-5223639 | CP001368.1 | (8) |
| Lysine-dependent acid resistance system (AR4) | cadA | Acid resistance | 5240905-5243052 | AE005174.2 | (8) |
| Lysine-dependent acid resistance system (AR4) | cadB | Acid resistance | 5243132-5244466 | AE005174.2 | (8) |
| Lysine-dependent acid resistance system (AR4) | cadC | Acid resistance | 5244832-5246370 | AE005174.2 | (8) |
| Response regulator | evgA | Stress response | 3281851-3282465 | AE005174.2 | (9) |
| Response regulator | evgS | Stress response | 3282470-3286063 | AE005174.2 | (9) |
| Non-LEE-encoded effector | espJ | Survival in Host | 2575299-2575817 | CP001368.1 | (10) |
| Glutamate-dependent acid resistance system (AR2) | gadA | Acid resistance | 4475543-4476943 | AE005174.2 | (8) |
| Glutamate-dependent acid resistance system (AR2) | gadB | Acid resistance | 1995182-1996582 | AE005174.2 | (8) |
| Glutamate-dependent acid resistance system (AR2) | gadC | Acid resistance | 2003776-2005311 | CP001368.1 | (8) |
| Glutamate-dependent acid resistance system (AR2) | gadE | Acid resistance | 4459619-4460146 | CP001368.1 | (8) |
| Glutamate-dependent acid resistance system (AR2) | gadW | Acid resistance | 4465143-4465871 | CP001368.1 | (8) |
| Glutamate-dependent acid resistance system (AR2) | gadX | Acid resistance | 4466239-4467063 | CP001368.1 | (8) |
| Quorum sensing | lsrA | Stress response | 2038989-2040524 | CP001368.1 | (11) |
| Quorum sensing | lsrB | Stress response | 2042550-2043572 | CP001368.1 | (11) |
| Quorum sensing | lsrC | Stress response | 2040518-2041546 | CP001368.1 | (11) |
| Quorum sensing | lsrD | Stress response | 2041546-2042538 | CP001368.1 | (11) |
| Quorum sensing | lsrE | Stress response | 2044845-2045603 | CP001368.1 | (11) |
| Quorum sensing | lsrF | Stress response | 2043599-2044474 | CP001368.1 | (11) |
| Quorum sensing | lsrG | Stress response | 2044498-2044788 | CP001368.1 | (11) |
| Quorum sensing | lsrK | Stress response | 2036116-2037708 | CP001368.1 | (11) |
| Quorum sensing | lsrR | Stress response | 2037787-2038740 | CP001368.1 | (11) |
| Quorum sensing | luxS | Stress response | 3597337-3597852 | CP001368.1 | (12) |
| Type III secreted effector proteins | nleA | Anti-inflammation | 1722107-1723432 | CP001368.1 | (13) |
| Type III secreted effector proteins | nleB1 | Anti-inflammation | 929579-930559 | CP001368.1 | (13) |
| Type III secreted effector proteins | nleB2 | Anti-inflammation | 3922737-3923726 | CP001368.1 | (13) |
| Type III secreted effector proteins | nleC | Anti-inflammation | 930620-931612 | CP001368.1 | (13) |
| Type III secreted effector proteins | nleD | Anti-inflammation | 933552-934250 | CP001368.1 | (13) |
| Type III secreted effector proteins | nleE | Anti-inflammation | 3923775-3924449 | CP001368.1 | (13) |
| Type III secreted effector proteins | nleH1 | Anti-inflammation | 932440-933321 | CP001368.1 | (13) |
| Type III secreted effector proteins | nleH2 | Anti-inflammation | 1725259-1726170 | CP001368.1 | (13) |
| Ornithine-dependent acid resistance system (AR5) | potE | Acid resistance | 795062-796381 | AE005174.2 | (8) |
| Quorum sensing | qseA | Stress response | 4191972-4192901 | AE005174.2 | (14) |
| Quorum sensing | qseB | Stress response | 3976571-3977230 | AE005174.2 | (14) |
| Quorum sensing | qseC | Stress response | 3977227-3978576 | AE005174.2 | (14, 15) |
| Quorum sensing | qseE | Stress response | 3479875-3481365 | AE005174.2 | (15) |
| Quorum sensing | qseF | Stress response | 3477673-3479007 | AE005174.2 | (16) |
| Quorum sensing | sdiA | Stress response | 2693592-2694314 | AE005174.2 | (12) |
| Ornithine-dependent acid resistance system (AR5) | speF | Acid resistance | 796378-798576 | AE005174.2 | (8) |
| Regulation of acid resistance | ydeO | Acid resistance | 2017749-2018510 | CP001368.1 | (17) |
| LEE-dependent Adherence | | | | | |
| Virulence factor | **Gene** | **Role** | **Genome POS** | **Accession No.** | **Reference** |
| Locus of Enterocyte Effacement | cesAB | Attachment and effacement of intestinal epithelial cells | 434205-434528 | ABHT01000001.1 | (18) |
| Locus of Enterocyte Effacement | cesD | Attachment and effacement of intestinal epithelial cells | 4672073-4672528 | CP001368.1 | (18) |
| Locus of Enterocyte Effacement | cesD2 | Attachment and effacement of intestinal epithelial cells | 1216736-1217143 | AERQ01000011.1 | (18) |
| Locus of Enterocyte Effacement | cesF | Attachment and effacement of intestinal epithelial cells | 4662052-4662435 | CP001368.1 | (18) |
| Locus of Enterocyte Effacement | cesL | Attachment and effacement of intestinal epithelial cells | 4668187-4668540 | CP001368.1 | (18) |
| Locus of Enterocyte Effacement | cesT | Attachment and effacement of intestinal epithelial cells | 4668305-4668775 | CP001368.1 | (18) |
| Locus of Enterocyte Effacement | eae | Attachment and effacement of intestinal epithelial cells | 4665441-4668245 | AE005174.2 | (18) |
| Locus of Enterocyte Effacement | ecsE | Attachment and effacement of intestinal epithelial cells | 2145144-2145362 | CP022689.1 | (18) |
| Locus of Enterocyte Effacement | escC | Attachment and effacement of intestinal epithelial cells | 4670521-4672059 | CP001368.1 | (18) |
| Locus of Enterocyte Effacement | escD | Attachment and effacement of intestinal epithelial cells | 4654119-4655339 | CP001368.1 | (18) |
| Locus of Enterocyte Effacement | escF | Attachment and effacement of intestinal epithelial cells | 4649517-4649738 | CP001368.1 | (18) |
| Locus of Enterocyte Effacement | escG | Attachment and effacement of intestinal epithelial cells | 4649233-4649511 | CP001368.1 | (18) |
| Locus of Enterocyte Effacement | escI | Attachment and effacement of intestinal epithelial cells | 4669057-4669485 | CP001368.1 | (18) |
| Locus of Enterocyte Effacement | escJ | Attachment and effacement of intestinal epithelial cells | 4669488-4670060 | CP001368.1 | (18) |
| Locus of Enterocyte Effacement | escK | Attachment and effacement of intestinal epithelial cells | 4677775-4678374 | CP001368.1 | (18) |
| Locus of Enterocyte Effacement | escL | Attachment and effacement of intestinal epithelial cells | 4677135-4677749 | CP001368.1 | (18) |
| Locus of Enterocyte Effacement | escN | Attachment and effacement of intestinal epithelial cells | 4664839-4666179 | CP001368.1 | (18) |
| Locus of Enterocyte Effacement | escO | Attachment and effacement of intestinal epithelial cells | 4664459-4664836 | CP001368.1 | (18) |
| Locus of Enterocyte Effacement | escP | Attachment and effacement of intestinal epithelial cells | 4664050-4664466 | CP001368.1 | (18) |
| Locus of Enterocyte Effacement | escQ | Attachment and effacement of intestinal epithelial cells | 4663170-4664087 | CP001368.1 | (18) |
| Locus of Enterocyte Effacement | escR | Attachment and effacement of intestinal epithelial cells | 432311-432964 | ABHT01000001.1 | (18) |
| Locus of Enterocyte Effacement | escS | Attachment and effacement of intestinal epithelial cells | 432042-43231 | ABHT01000001.1 | (18) |
| Locus of Enterocyte Effacement | escT | Attachment and effacement of intestinal epithelial cells | 431266-432042 | ABHT01000001.1 | (18) |
| Locus of Enterocyte Effacement | escU | Attachment and effacement of intestinal epithelial cells | 430236-431273 | ABHT01000001.1 | (18) |
| Locus of Enterocyte Effacement | escV | Attachment and effacement of intestinal epithelial cells | 4666163-4668190 | CP001368.1 | (18) |
| Locus of Enterocyte Effacement | espA | Attachment and effacement of intestinal epithelial cells | 4652284-4652862 | CP001368.1 | (18) |
| Locus of Enterocyte Effacement | espB | Attachment and effacement of intestinal epithelial cells | 4650188-4651126 | CP001368.1 | (18) |
| Locus of Enterocyte Effacement | espD | Attachment and effacement of intestinal epithelial cells | 4651147-4652271 | CP001368.1 | (18) |
| Locus of Enterocyte Effacement | espF | Attachment and effacement of intestinal epithelial cells | 4648402-4649148 | CP001368.1 | (18) |
| Locus of Enterocyte Effacement | espG | Attachment and effacement of intestinal epithelial cells | 4680565-4681761 | CP001368.1 | (18) |
| Locus of Enterocyte Effacement | espH | Attachment and effacement of intestinal epithelial cells | 4662633-4663139 | CP001368.1 | (18) |
| Locus of Enterocyte Effacement | espZ | Attachment and effacement of intestinal epithelial cells | 4668725-4669024 | CP001368.1 | (18) |
| Locus of Enterocyte Effacement | etgA | Attachment and effacement of intestinal epithelial cells | 4673947-4674405 | CP001368.1 | (18) |
| Locus of Enterocyte Effacement | grlA | Attachment and effacement of intestinal epithelial cells | 4672912-4673325 | CP001368.1 | (18) |
| Locus of Enterocyte Effacement | grlR | Attachment and effacement of intestinal epithelial cells | 4673380-4673751 | CP001368.1 | (18) |
| Locus of Enterocyte Effacement | map | Attachment and effacement of intestinal epithelial cells | 4661175-4661786 | CP001368.1 | (18) |
| Locus of Enterocyte Effacement | ler | Attachment and effacement of intestinal epithelial cells | - | AF328682.1 | (18) |
| Locus of Enterocyte Effacement | rorf1 | Attachment and effacement of intestinal epithelial cells | 4681889-4682707 | CP001368.1 | (18) |
| Locus of Enterocyte Effacement | sepD | Attachment and effacement of intestinal epithelial cells | 4670066-4670521 | CP001368.1 | (18) |
| Locus of Enterocyte Effacement | sepL | Attachment and effacement of intestinal epithelial cells | 4652921-4653976 | CP001368.1 | (18) |
| Locus of Enterocyte Effacement | tir | Attachment and effacement of intestinal epithelial cells | 4668913-4670589 | AE005174.2 | (18) |
| Fucose sensing | z0462 | Sensor kinase | 443763-445304 | AE005174.2 | (19) |
| Fucose response | z0643 | Response regulator | 445304-445933 | AE005174.2 | (19) |
| LEE-independent Adherence | | | | | |
| Virulence factor | **Gene** | **Role** | **Genome POS** | **Accession No.** | **Reference** |
| Calcium-binding antigen 43 homologous | cah | Autotransporter | 1388023-1390872 | CP001368.1 | (20) |
| Enterohaemorrhagic E. coli autotransporters | ehaA | Autotransporter | 379670-383719 | AE005174.2 | (21) |
| Enterohaemorrhagic E. coli autotransporters | ehaB | Autotransporter | 451257-454199 | AE005174.2 | (22) |
| Enterohaemorrhagic E. coli autotransporters | ehaD | Autotransporter | 3567614-3572200 | AE005174.2 | (23) |
| Haemorrhagic coli pilus | hcpA | Pilus | 121579-122019 | CP001368.1 | (24) |
| Adhesin | iha | Adhesin | 1362242-1364332 | CP001368.1 | (25) |
| Long polar fimbriae | lpfA | Fimbriae | 4477159-4477695 | CP010304.1 | (26) |
| Long polar fimbriae | lpfA2 | Fimbriae | 4723620-4724222 | CP010304.1 | (27) |
| Long polar fimbriae | lpfB | Fimbriae | 4476408-4477097 | CP010304.1 | (26) |
| Long polar fimbriae | lpfB2 | Fimbriae | 4723133-4723516 | CP010304.1 | (27) |
| Long polar fimbriae | lpfC | Fimbriae | 4475276-4476379 | CP010304.1 | (26) |
| Long polar fimbriae | lpfC2' | Fimbriae | 4722831-4722992 | CP010304.1 | (26-28) |
| Long polar fimbriae | lpfC2 | Fimbriae | 4720275-4722809 | CP010304.1 | (26-28) |
| Long polar fimbriae | lpfD' | Fimbriae | 4472736-4473791 | CP010304.1 | (26-28) |
| Long polar fimbriae | lpfD | Fimbriae | 4718083-4719165 | CP010304.1 | (26-28) |
| Long polar fimbriae | lpfD2 | Fimbriae | 4719193-4720263 | CP010304.1 | (26-28) |
| Long polar fimbriae | lpfE | Fimbriae | 4472201-4472731 | CP010304.1 | (26-28) |
| Serine protease | pssA | Secreted serine protease | 3512760-3514118 | AE005174.2 | (29) |
| E. coli common pilus | yagZ | Pilus | 342493-343080 | AE005174.2 | (30) |
| Putative laminin-binding fimbriae | ycbQ | Fimbriae | 1224337-1224885 | CP001846.1 | (31) |
| Putative laminin-binding fimbriae | ycbR | Fimbriae | 1224968-1225669 | CP001846.1 | (31) |
| Putative laminin-binding fimbriae | ycbS(pseudo) | Fimbriae | 1225694-1228293 | CP001846.1 | (31) |
| Putative laminin-binding fimbriae | ycbT | Fimbriae | 1228284-1229354 | CP001846.1 | (31) |
| pO157 Plasmid | | | | | |
| Virulence factor | **Gene** | **Role** | **Genome POS** | **Accession No.** | **Reference** |
| Eae gene-positive Conserved Fragments | ecf1 | Adhesin | 3695-4516 | AF043470.1 | (32) |
| Eae gene-positive Conserved Fragments | ecf2 | Adhesin | 2589-3695 | AF043470.1 | (32) |
| Eae gene-positive Conserved Fragments | ecf3 | Adhesin | 751-2499 | AF043470.1 | (32) |
| Eae gene-positive Conserved Fragments | ecf4 | Adhesin | 1-705 | AF043470.1 | (32) |
| Enterohaemolysin | ehxA | Lysis of red blood cells | 39822-42818 | AF074613.1 | (33) |
| Enterohaemolysin | ehxB | Lysis of red blood cells | 42868-44988 | AF074613.1 | (33) |
| Enterohaemolysin | ehxC | Lysis of red blood cells | 39305-39820 | AF074613.1 | (33) |
| Enterohaemolysin | ehxD | Lysis of red blood cells | 44992-46431 | AF074613.1 | (33) |
| Serine protease | espP | Autotransporter | 11242-15144 | AF074613.1 | (34) |
| Type II secretion system | etpC | Type II secretion system | 25799-26674 | AF074613.1 | (35) |
| Type II secretion system | etpD | Type II secretion system | 26714-28642 | AF074613.1 | (35) |
| Type II secretion system | etpE | Type II secretion system | 28642-30147 | AF074613.1 | (35) |
| Type II secretion system | etpF | Type II secretion system | 30149-31372 | AF074613.1 | (35) |
| Type II secretion system | etpG | Type II secretion system | 31403-31837 | AF074613.1 | (35) |
| Type II secretion system | etpH | Type II secretion system | 31834-32388 | AF074613.1 | (35) |
| Type II secretion system | etpI | Type II secretion system | 32385-32750 | AF074613.1 | (35) |
| Type II secretion system | etpJ | Type II secretion system | 32747-33346 | AF074613.1 | (35) |
| Type II secretion system | etpK | Type II secretion system | 33343-34320 | AF074613.1 | (35) |
| Type II secretion system | etpL | Type II secretion system | 34254-35531 | AF074613.1 | (35) |
| Type II secretion system | etpM | Type II secretion system | 35518-36030 | AF074613.1 | (35) |
| Type II secretion system | etpN | Type II secretion system | 36088-36921 | AF074613.1 | (35) |
| Type II secretion system | etpO | Type II secretion system | 37013-37414 | AF074613.1 | (35) |
| Catalase-peroxidase | katP | Adhesin | 7373-9583 | AF074613.1 | (36) |
| C1 Esterase Inhibitor | stcE | Adhesin, inflammation | 60443-63103 | AF074613.1 | (37) |
| Toxin B | toxB | Adhesin | 39533-49042 | AF074613.1 | (38) |

1. Baines SL, Gonçalves da Silva A, Carter G, Jennison AV, Rathnayake I, Graham RM, et al. Complete microbial genomes for public health in Australia and Southwest Pacific. bioRxiv. 2019:829663.

2. Franz E, Rotariu O, Lopes BS, MacRae M, Bono JL, Laing C, et al. Phylogeographic Analysis Reveals Multiple International transmission Events Have Driven the Global Emergence of Escherichia coli O157:H7. Clinical infectious diseases : an official publication of the Infectious Diseases Society of America. 2019;69(3):428-37.

3. Pintara AP, Guglielmino CJD, Rathnayake IU, Huygens F, Jennison AV. Molecular Prediction of the O157:H-Negative Phenotype Prevalent in Australian Shiga Toxin-Producing Escherichia coli Cases Improves Concordance of In Silico Serotyping with Phenotypic Motility. Journal of clinical microbiology. 2018;56(4).

4. Ingle DJ, Gonçalves da Silva A, Valcanis M, Ballard SA, Seemann T, Jennison AV, et al. Emergence and divergence of major lineages of Shiga-toxin-producing Escherichia coli in Australia. Microbial genomics. 2019;5(5).

5. Barth SA, Menge C, Eichhorn I, Semmler T, Wieler LH, Pickard D, et al. The Accessory Genome of Shiga Toxin-Producing Escherichia coli Defines a Persistent Colonization Type in Cattle. Appl Environ Microbiol. 2016;82(17):5455-64.

6. Rogers MT, Zimmerman R, Scott ME. Histone-like nucleoid-structuring protein represses transcription of the ehx operon carried by locus of enterocyte effacement-negative Shiga toxin-expressing Escherichia coli. Microbial pathogenesis. 2009;47(4):202-11.

7. Melton-Celsa AR. Shiga Toxin (Stx) Classification, Structure, and Function. Microbiology spectrum. 2014;2(2).

8. Aquino P, Honda B, Jaini S, Lyubetskaya A, Hosur K, Chiu JG, et al. Coordinated regulation of acid resistance in Escherichia coli. BMC Systems Biology. 2017;11.

9. Nishino K, Inazumi Y, Yamaguchi A. Global analysis of genes regulated by EvgA of the two-component regulatory system in Escherichia coli. J Bacteriol. 2003;185(8):2667-72.

10. Dahan S, Wiles S, La Ragione RM, Best A, Woodward MJ, Stevens MP, et al. EspJ is a prophage-carried type III effector protein of attaching and effacing pathogens that modulates infection dynamics. Infection and immunity. 2005;73(2):679-86.

11. Bansal T, Englert D, Lee J, Hegde M, Wood TK, Jayaraman A. Differential effects of epinephrine, norepinephrine, and indole on Escherichia coli O157:H7 chemotaxis, colonization, and gene expression. Infection and immunity. 2007;75(9):4597-607.

12. Park H, Lee K, Yeo S, Shin H, Holzapfel WH. Autoinducer-2 Quorum Sensing Influences Viability of Escherichia coli O157:H7 under Osmotic and In Vitro Gastrointestinal Stress Conditions. Front Microbiol. 2017;8.

13. Pearson JS, Hartland EL. The Inflammatory Response during Enterohemorrhagic Escherichia coli Infection. Microbiology spectrum. 2014;2(4):Ehec-0012-2013.

14. Sperandio V, Li CC, Kaper JB. Quorum-Sensing Escherichia coli Regulator A: a Regulator of the LysR Family Involved in the Regulation of the Locus of Enterocyte Effacement Pathogenicity Island in Enterohemorrhagic E. coli. Infection and immunity. 2002;70(6):3085-93.

15. Njoroge J, Sperandio V. Enterohemorrhagic Escherichia coli virulence regulation by two bacterial adrenergic kinases, QseC and QseE. Infection and immunity. 2012;80(2):688-703.

16. Parker CT, Russell R, Njoroge JW, Jimenez AG, Taussig R, Sperandio V. Genetic and Mechanistic Analyses of the Periplasmic Domain of the Enterohemorrhagic Escherichia coli QseC Histidine Sensor Kinase. J Bacteriol. 2017;199(8).

17. Masuda N, Church GM. Regulatory network of acid resistance genes in Escherichia coli. Molecular microbiology. 2003;48(3):699-712.

18. Gaytán MO, Martínez-Santos VI, Soto E, González-Pedrajo B. Type Three Secretion System in Attaching and Effacing Pathogens. Frontiers in Cellular and Infection Microbiology. 2016;6.

19. Pacheco AR, Curtis MM, Ritchie JM, Munera D, Waldor MK, Moreira CG, et al. Fucose sensing regulates bacterial intestinal colonization. Nature. 2012;492(7427):113-7.

20. Torres AG, Perna NT, Burland V, Ruknudin A, Blattner FR, Kaper JB. Characterization of Cah, a calcium-binding and heat-extractable autotransporter protein of enterohaemorrhagic Escherichia coli. Molecular microbiology. 2002;45(4):951-66.

21. Wells TJ, Sherlock O, Rivas L, Mahajan A, Beatson SA, Torpdahl M, et al. EhaA is a novel autotransporter protein of enterohemorrhagic Escherichia coli O157:H7 that contributes to adhesion and biofilm formation. Environmental microbiology. 2008;10(3):589-604.

22. Wells TJ, McNeilly TN, Totsika M, Mahajan A, Gally DL, Schembri MA. The Escherichia coli O157:H7 EhaB autotransporter protein binds to laminin and collagen I and induces a serum IgA response in O157:H7 challenged cattle. Environmental microbiology. 2009;11(7):1803-14.

23. Easton D, Totsika M, Allsopp L, Phan M-D, Idris A, Wurpel D, et al. Characterization of EhaJ, a New Autotransporter Protein from Enterohemorrhagic and Enteropathogenic Escherichia coli. Frontiers in Microbiology. 2011;2(120).

24. Xicohtencatl-Cortes J, Monteiro-Neto V, Ledesma MA, Jordan DM, Francetic O, Kaper JB, et al. Intestinal adherence associated with type IV pili of enterohemorrhagic Escherichia coli O157:H7. The Journal of clinical investigation. 2007;117(11):3519-29.

25. Tarr PI, Bilge SS, Vary JC, Jr., Jelacic S, Habeeb RL, Ward TR, et al. Iha: a novel Escherichia coli O157:H7 adherence-conferring molecule encoded on a recently acquired chromosomal island of conserved structure. Infection and immunity. 2000;68(3):1400-7.

26. Torres AG, Giron JA, Perna NT, Burland V, Blattner FR, Avelino-Flores F, et al. Identification and characterization of lpfABCC'DE, a fimbrial operon of enterohemorrhagic Escherichia coli O157:H7. Infection and immunity. 2002;70(10):5416-27.

27. Torres AG, Kanack KJ, Tutt CB, Popov V, Kaper JB. Characterization of the second long polar (LP) fimbriae of Escherichia coli O157:H7 and distribution of LP fimbriae in other pathogenic E. coli strains. FEMS microbiology letters. 2004;238(2):333-44.

28. Cordonnier C, Etienne-Mesmin L, Thevenot J, Rougeron A, Renier S, Chassaing B, et al. Enterohemorrhagic Escherichia coli pathogenesis: role of Long polar fimbriae in Peyer's patches interactions. Scientific reports. 2017;7:44655.

29. van Diemen PM, Dziva F, Stevens MP, Wallis TS. Identification of enterohemorrhagic Escherichia coli O26:H- genes required for intestinal colonization in calves. Infection and immunity. 2005;73(3):1735-43.

30. Rendon MA, Saldana Z, Erdem AL, Monteiro-Neto V, Vazquez A, Kaper JB, et al. Commensal and pathogenic Escherichia coli use a common pilus adherence factor for epithelial cell colonization. Proceedings of the National Academy of Sciences of the United States of America. 2007;104(25):10637-42.

31. Samadder P, Xicohtencatl-Cortes J, Saldana Z, Jordan D, Tarr PI, Kaper JB, et al. The Escherichia coli ycbQRST operon encodes fimbriae with laminin-binding and epithelial cell adherence properties in Shiga-toxigenic E. coli O157:H7. Environmental microbiology. 2009;11(7):1815-26.

32. Yoon JW, Minnich SA, Ahn JS, Park YH, Paszczynski A, Hovde CJ. Thermoregulation of the Escherichia coli O157:H7 pO157 ecf operon and lipid A myristoyl transferase activity involves intrinsically curved DNA. Molecular microbiology. 2004;51(2):419-35.

33. Schmidt H, Beutin L, Karch H. Molecular analysis of the plasmid-encoded hemolysin of Escherichia coli O157:H7 strain EDL 933. Infection and immunity. 1995;63(3):1055-61.

34. Brunder W, Schmidt H, Karch H. EspP, a novel extracellular serine protease of enterohaemorrhagic Escherichia coli O157:H7 cleaves human coagulation factor V. Molecular microbiology. 1997;24(4):767-78.

35. Schmidt H, Henkel B, Karch H. A gene cluster closely related to type II secretion pathway operons of gram-negative bacteria is located on the large plasmid of enterohemorrhagic Escherichia coli O157 strains. FEMS microbiology letters. 1997;148(2):265-72.

36. Brunder W, Schmidt H, Karch H. KatP, a novel catalase-peroxidase encoded by the large plasmid of enterohaemorrhagic Escherichia coli O157:H7. Microbiology (Reading, England). 1996;142 ( Pt 11):3305-15.

37. Lathem WW, Grys TE, Witowski SE, Torres AG, Kaper JB, Tarr PI, et al. StcE, a metalloprotease secreted by Escherichia coli O157:H7, specifically cleaves C1 esterase inhibitor. Molecular microbiology. 2002;45(2):277-88.

38. Tatsuno I, Horie M, Abe H, Miki T, Makino K, Shinagawa H, et al. toxB gene on pO157 of enterohemorrhagic Escherichia coli O157:H7 is required for full epithelial cell adherence phenotype. Infection and immunity. 2001;69(11):6660-9.
